# Effect of Water Deficit on Germination, Growth and Biochemical Responses of Four Potentially Invasive Ornamental Grass Species

**DOI:** 10.3390/plants12061260

**Published:** 2023-03-10

**Authors:** Diana M. Mircea, Elena Estrelles, Mohamad Al Hassan, Pilar Soriano, Radu E. Sestras, Monica Boscaiu, Adriana F. Sestras, Oscar Vicente

**Affiliations:** 1 Department of Forestry, University of Agricultural Sciences and Veterinary Medicine Cluj-Napoca, 3-5 Manastur Street, 400372 Cluj-Napoca, Romania; diana-maria.mircea@usamvcluj.ro; 2 Institute for the Conservation and Improvement of Valencian Agrodiversity (COMAV), Universitat Politècnica de València, Camino de Vera s/n, 46022 Valencia, Spain; 3 Cavanilles Institute of Biodiversity and Evolutionary Biology, Botanical Garden, University of Valencia, Quart, 80, 46008 Valencia, Spain; elena.estrelles@uv.es (E.E.); pilar.soriano@uv.es (P.S.); 4 Laboratory of Plant Breeding, Wageningen University and Research (WUR), Droevendaalsesteeg 1, 6708 PB Wageningen, The Netherlands; mohamed.alhassan@wur.nl; 5 Department of Plant Sciences, Aeres University of Applied Sciences, 8251 JZ Dronten, The Netherlands; 6 Department of Horticulture and Landscape, University of Agricultural Sciences and Veterinary Medicine Cluj-Napoca, 3-5 Manastur Street, 400372 Cluj-Napoca, Romania; rsestras@usamvcluj.ro; 7 Mediterranean Agroforestry Institute (IAM), Universitat Politècnica de València, Camino de Vera s/n, 46022 Valencia, Spain; mobosnea@eaf.upv.es

**Keywords:** abiotic stress, seed germination, invasive potential, ornamental grasses, stress biomarkers, climate change

## Abstract

Ornamental plant species introduced into new environments can exhibit an invasive potential and adaptability to abiotic stress factors. In this study, the drought stress responses of four potentially invasive ornamental grass species (*Cymbopogon citratus*, *Cortaderia selloana*, *Pennisetum alopecuroides* and *P. setaceum*) were analysed. Several seed germination parameters were determined under increasing polyethylene glycol (PEG 6000) concentrations. Additionally, plants in the vegetative stage were subjected to intermediate and severe water stress treatments for four weeks. All species registered high germination rates in control conditions (no stress treatment), even at high PEG concentrations, except *C. citratus*, which did not germinate at −1 MPa osmotic potential. Upon applying the water stress treatments, *P. alopecuroides* plants showed the highest tolerance, and *C. citratus* appeared the most susceptible to drought. Stress-induced changes in several biochemical markers (photosynthetic pigments, osmolytes, antioxidant compounds, root and shoot Na^+^ and K^+^ contents), highlighted different responses depending on the species and the stress treatments. Basically, drought tolerance seems to depend to a large extent on the active transport of Na^+^ and K^+^ cations to the aerial part of the plants, contributing to osmotic adjustment in all four species and, in the case of the most tolerant *P. alopecuroides*, on the increasing root K^+^ concentration under water deficit conditions. The study shows the invasive potential of all species, except *C. citratus*, in dry areas such as the Mediterranean region, especially in the current climate change scenario. Particular attention should be given to *P. alopecuroides*, which is widely commercialised in Europe as ornamental.

## 1. Introduction

Most crops and ornamental plants cultivated in different regions of the world are not native to these territories [1]. A small fraction of these species, especially those introduced for ornamental purposes, may escape human control and become invasive. An alien species is naturalised when it can form self-sustaining populations in a new territory but becomes invasive only when it overcomes abiotic and biotic barriers and spreads far from the original introduction site [2,3]. Biological invasions represent one of the greatest threats to natural environments, second only to habitat loss [4]. The presence of invasive plant species generates strong competition for resources and a change in the composition and diversity of the aerial and subterranean communities [3,5,6,7], such as modifications of abiotic and biotic conditions [8], changes in hydrologic cycles [9], increased frequency and intensity of disturbance cycles [10,11] and, in general, a reduction in ecosystem services [12]. A major source of invasive species is horticulture, which is responsible for the deliberate introduction of about half of the invasive species in the United States [13]. The invasive potential of horticultural plants is enhanced by some of their traits such as long flowering time, low maintenance requirements and wide adaptability, which improve plant overall performance even under suboptimal conditions [14,15].

As ornamental horticulture has to face strict restrictions on the import of potentially invasive species [1], the assessment of invasion risk is evolving and becoming more extended. A plethora of studies indicate that global warming is favouring biological invasions, not only by changing climatic barriers but also by increasing the competitiveness of alien species under the new climatic conditions [16,17]. One clear consequence of climate change is an increase in the intensity, frequency and duration of drought periods in many areas of the world [18], causing a loss of biodiversity due to habitat loss, migration of local species and the spread of invasive alien ones, especially since the latter are better adapted to environmental changes [19,20]. In contrast, several reports have challenged this widespread assumption, e.g., [21], and others have linked the observed changes in invasiveness potential with the taxonomy or the profile of the invaded habitat [22].

This study aims to provide evidence of the effects of water deficiency on the invasion potential of four ornamental grass species of the Poaceae family. The investigated taxa, *Cymbopogon citratus* (DC.) Stapf, *Cortaderia selloana* (Schult. & Schult.f.) Asch. & Graebn., *Pennisetum alopecuroides* (L.) Spreng., and *Pennisetum setaceum* (Forssk.) Chiov, have different native areas and ecological optima. All four are perennial grasses with documented invasiveness potential [23,24,25,26,27]; only *C. selloana* uses C3 carbon fixation, whereas *C. citratus* and both *Pennisetum* are C4 plant species.

**Figure 1:**
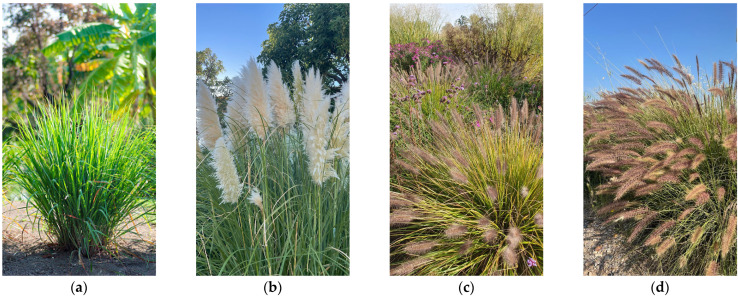
The species analysed in the study: (**a**) *Cymbopogon citratus*, (**b**) *Cortaderia selloana*, (**c**) *Pennisetum alopecuroides*, and (**d**) *Pennisetum setaceum*.

*Cymbopogon citratus*, lemongrass (Figure 1a), is a tufted aromatic herb native to India and Sri Lanka [28] that thrives in tropical and subtropical areas in South Africa and Central America [29]. Lemongrass is one of the most widely grown essential oil plants in many tropical and subtropical regions, used in the pharmaceutical, food and cosmetics industries [30]. It has an optimal growth in warm climates with a rainfall of 2500–3000 mm yearly [31]. Its growth has been reported to decrease under water deficit [32], but its essential oil yield was enhanced by moderate water stress [32,33]. The species has been also reported as tolerant to mild soil salinity up to 80 mM NaCl [34]. Given its high adaptability and vigorous growth, it is currently listed as a weed in Mexico and an invasive species in St. Lucia Island [35]. The pampas grass, *Cortaderia selloana*, originating from South America, is herbaceous, growing up to 3 m tall, with robust tillers (Figure 1b); it grows on moist soils in grassland plains, sparse shrublands and riverine habitats [27]. The species is usually found in humid settings in Mediterranean regions that experience water deficits in summer [36]; however, it can adapt to water stress as shown by the rise in the root–shoot ratio under moderate and severe water stress [37]. Pampas grass was reported as resilient to moderate water deficit and heat stress [38,39] and to have the broadest range of niches among other monocotyledons found in marshes and coastal environments [40,41].

The *Pennisetum* species are tolerant to water stress [42] and are very popular as ornamentals due to their foliage and attractive flower spikes. Some species are used as forage or have been proposed for the reclamation of salinised areas due to their relative salt tolerance [43]. However, many species of this genus are included in the list of noxious invasives, such as *P. setaceum*, *P. purpureum*, *P. villosum* and *P. clandestinum* [27,44]. Two of them were included in this study. *Pennisetum alopecuroides*, Chinese fountain grass (Figure 1c), a native species from temperate regions of East Asia and western Australia [45], has been recently reported as potential invasive in the USA [23,26]. *Pennisetum setaceum*, commonly known as fountain grass (Figure 1d), with origin in NE Africa, has been largely used for soil stabilisation, but became a recognised dangerous invasive species, successfully invading different types of habitats, from tropical or subtropical, to arid and semi-arid regions [24]. Regarding their abiotic stress tolerance, *P. alopecuroides* was reported as drought tolerant [46] and moderate [47], and even highly salt tolerant and recommended for soils desalinisation [48]. *P. setaceum* has also long been known to have a wide ecological amplitude [49], which makes it well suited for colonising frequently disturbed sites with fluctuating resource availability or irregular rainfall pattern [50].

The objectives of this work were: (i) to evaluate the responses of the four species under conditions of osmotic stress during seed germination and to water deficit during vegetative growth; (ii) to analyse possible tolerance mechanisms; (iii) to assess their invasive potential, and range extension possibilities; and (iv) to detect specific traits in the most invasive species.

## 2. Results

### 2.1. Seed Germination and Recovery of Germination

**Figure 2:**
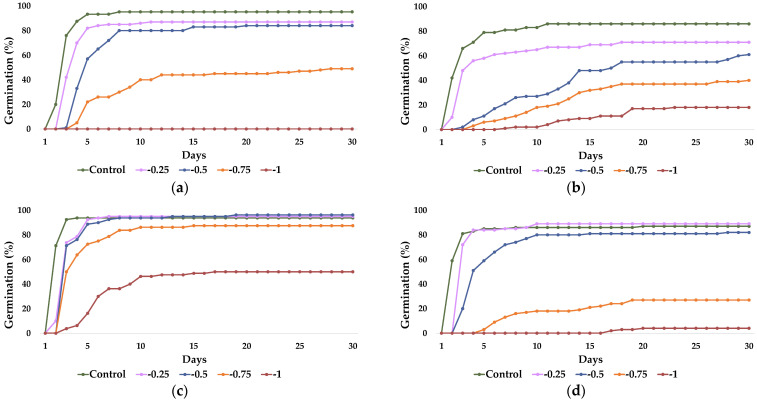
Evolution of seed germination over 30 days of osmotic stress treatments using PEG at the indicated concentrations, expressed as osmotic potential (MPa), in (**a**) *C. citratus*, (**b**) *C. selloana*, (**c**) *P. alopecuroides*, and (**d**) *P. setaceum*. Means of cumulative germination percentages of four technical replicas (plates) are presented for each treatment and species. Control: germination in distilled water.

In all four studied species, germination percentages and velocity were detrimentally and progressively affected by increasing polyethylene glycol (PEG) concentrations (Figure 2). *Cortaderia selloana* (Figure 2b) and *P. alopecuroides* (Figure 2c) seeds maintained certain levels of germination under the highest applied PEG concentration (−1 MPa), whereas in *C. citratus* (Figure 2a) and *P. setaceum* (Figure 2d), seed germination was almost or completely inhibited, under the same osmotic potentials. Under control conditions (distilled water), maximum germination percentages were reached by the 8th day after sowing in *C. citratus* (95.3%, Figure 2a), after 11 days in *C. selloana* (86%, Figure 2b), 4 days in *P. alopecuroides* (94%, Figure 2c), or 20 days in *P. setaceum* (87%, Figure 2d).

**Figure 3:**
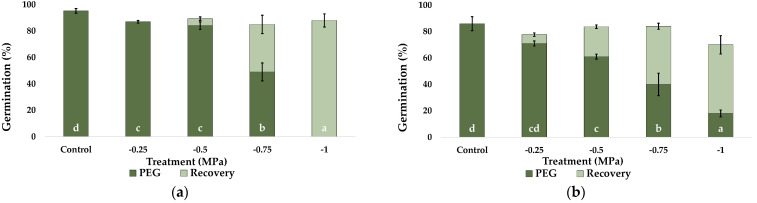

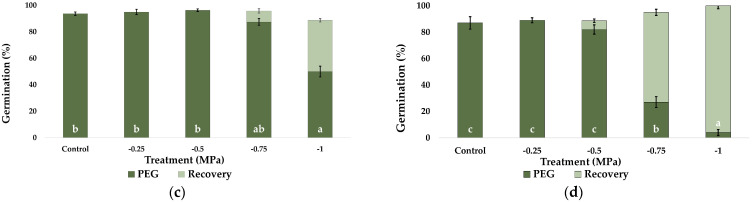
Final germination percentages after 30 days under increasing concentrations of PEG, as indicated (dark green), and germination recovery percentages, 20 days after transferring previously stressed, not germinated seeds to control conditions (light green) for: (**a**) *C. citratus*, (**b**) *C. selloana*, (**c**) *P. alopecuroides*, and (**d**) *P. setaceum*. Control: germination in distilled water. Values shown are means ± SE (*n* = 4). Different lowercase letters within the bars indicate significant differences between treatments, for each species, according to the Tukey post hoc test (*p* < 0.05).

Upon PEG application for 30 days, the final germination percentages decreased in all four investigated species (Figure 3). However, their sensitivity to osmotic stress varied, with *P. alopecuroides* seeds being the most tolerant (Figure 3c). Up to −0.5 MPa PEG, the highest germination percentage seen in the control was maintained; however, −0.75 MPa caused it to be somewhat lowered (10%), and −1 MPa caused it to drop to roughly 50%. For the other three species, final germination percentages decreased gradually in parallel with increasing PEG concentrations. This reduction was modest in *C. citratus* (Figure 3a) and *P. setaceum* (Figure 3d) at −0.25 and −0.5 MPa, but it was considerable at −0.75 MPa (55% and 70%, respectively).

The highest PEG concentration tested (−1 MPa) caused a complete (*C. citratus*) or very strong (down to 4%, *P. setaceum*) germination inhibition. These values decreased more progressively in *C. selloana*, from the control’s 86% to 70, 60, 40 and 20%, at −0.25, −0.5, −0.75 and −1 MPa, respectively (Figure 3b).

The recovery ability of the seeds that failed to germinate in the presence of PEG was tested by placing them in new Petri dishes with distilled water for 20 days (Figure 3). Full recovery was observed in the four species, with final germination percentages equivalent to, or even exceeding, those registered in the control treatments. For instance, in *P. setaceum*, the recovery treatment of −0.75 MPa and −1 MPa stressed seeds resulted in final germination percentages of 93 and 96%, respectively, compared to 87% in the control (Figure 3d).

**Figure 4:**
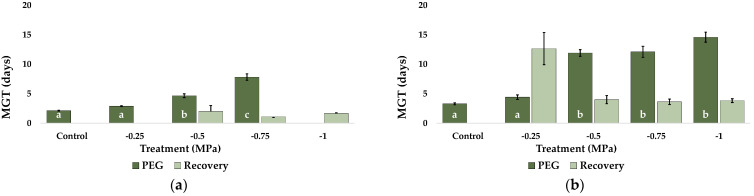

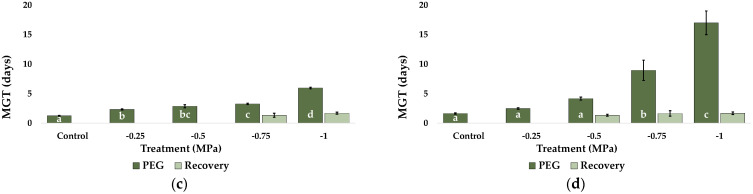
Mean Germination Time (MGT) of seeds of the four studied species after 30 days of the indicated PEG treatments (dark green), and after 20 days of “recovery” (light green). MGT in: (**a**) *C. citratus*, (**b**) *C. selloana*, (**c**) *P. alopecuroides*, and (**d**) *P. setaceum*. Control: germination in distilled water Values shown are means ± SE (*n* = 4). Different lowercase letters within the bars indicate significant differences between treatments, for each species, according to the Tukey post hoc test (*p* < 0.05).

Mean germination time (MGT) was calculated during the stress and subsequent recovery assays (Figure 4). Overall, MGT increased with increasing PEG concentrations. In *C. citratus* and both *Pennisetum* species, MGT rose progressively in the initial germination assays; in the recovery assays, for all previously stressed seeds, germination time was very short once optimal conditions were restored (Figure 4a,c,d). On the other hand, *C. selloana* seeds showed a somewhat different pattern, with a significant MGT increase at −0.5 MPa, which did not vary at higher PEG concentrations; in this case, MGT in the recovery treatments were also lower than in the corresponding stress treatments, except for the seeds that did not germinate in the presence of PEG at −0.25 MPa (Figure 4b).

Other germination parameters (reduction in germination percentage; first germination day; last germination day; time spread germination; the speed of emergence; germination index) were also calculated to better show differences between the studied species and are detailed in Table S1 of Supplementary Materials.

**Figure 5:**
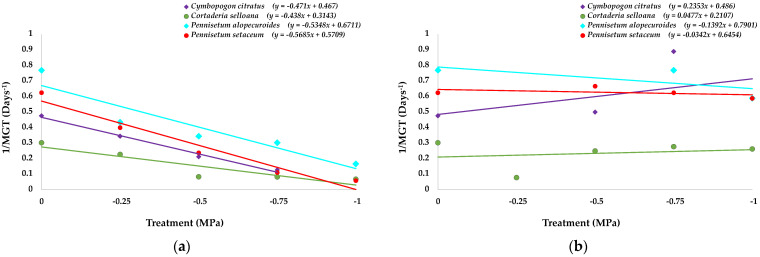
Germination velocity (1/MGT), in relation to the osmotic potential of the germination medium, generated by PEG addition, for the four analysed species, (**a**) for the PEG-treated seeds in the 30-day initial germination tests, (**b**) after 20 days of “recovery”. Control: germination in distilled water.

The graph in Figure 5a shows the trend lines of the variation in MGT against the decrease in water potential caused by the increase in the PEG 6000 concentration for the four species. The germination speed reduction pattern under osmotic stress is similar for all of them except for *C. selloana*, which does not follow the general trend, especially at the highest PEG concentrations. There is a fraction of seeds that maintain the germination rate practically invariable from −0.5 to −1 MPa, as MGT values do not show statistically significant differences (Figure 4b), even though the germination percentage is significantly reduced under these conditions (Figure 3b).

Considering the competitiveness concept in relation to the germination velocity, the most competitive species was *P. alopecuroides*, even at the lowest osmotic potentials. On the contrary, *C. selloana* was the least competitive, at least up to −0.75 MPa, as its line is below the others (Figure 5a). At the lowest osmotic potential values, this trend changes, and this species increases its competitiveness, becoming the second most competitive as germination velocity stabilises and the line slope decreases. These results agree with values of the reduction in germination percentage (RGP) (Table S1), as *C. selloana* is the second species with the lowest RGP at the lowest osmotic potentials.

Figure 5b shows the effect of previous PEG exposure on non-germinated seeds after they were transferred to distilled water. Seed germination recovery did not indicate any priming effect of the osmotic stress during the initial germination tests, as the MGT was not significantly different from the corresponding control, for any of the four analysed species (*p* < 0.05).

The calculated parameters obtained when applying the hydrotime model are presented in Table 1. These parameters are related to the effect of osmotic potential decreasing the seed’s germination rate. Values of the base osmotic potential for maximum germination (Ψb) varied between the species, from −0.7 MPa to −1.3 MPa. The species in decreasing order are *C. citratus*, *P. setaceum*, *C. selloana* and *P. alopecuroides*. When considering the water potential for 50% inhibition (Ψb_50_), the obtained values ranged between −1.1 MPa and −2.6 MPa, with *C. selloana* showing the highest value, followed by *P. setaceum*, *C. citratus* and *P. alopecuroides*, which had the lowest Ψb_50_ value.

With respect to the hydrotime (θ) (Table 1), the highest value was calculated for *C. selloana* as this species shows the slowest germination response, whereas both species of *Pennisetum* had the lowest values, corresponding to the fastest response.

### 2.2. Seedling Analysis

Osmotic stress affected seed germination morphologically, causing a reduction in seedling’s radicle and hypocotyl lengths in parallel to increasing PEG concentrations (Table 2). Under control conditions, *C. selloana* seedlings had a radicle significantly shorter (4.4 mm) than the other three investigated species (ca. 20 mm). However, in relative terms, they showed the smallest reduction in length under stress compared with the control (Table 2).

Similarly, the hypocotyls of *C. selloana* seedlings were shorter than those of the other species. Hypocotyl elongation was strongly reduced in parallel to increasing PEG concentrations in all taxa, in this case also including *C. selloana*. Thus, at −1 MPa, a reduction of about 90% in the control value was calculated for *C. selloana* and *P. alopecuroides*, with an even higher reduction, almost 98%, for *P. setaceum* (Table 2). As mentioned above, *C. citratus* seeds did not germinate at this level of osmotic stress. Accordingly, seed germination vigour dropped in response to the applied stress treatments to very low values when the highest PEG concentration was used. For control seeds germinating in water, those of *P. setaceum* were the most vigorous, followed by *P. alopecuroides* and *C. citratus*, and then finally *C. selloana*, with a seed vigour index (SVI) of about 30% of that of *P. setaceum*.

### 2.3. Plant Growth

**Figure 6:**
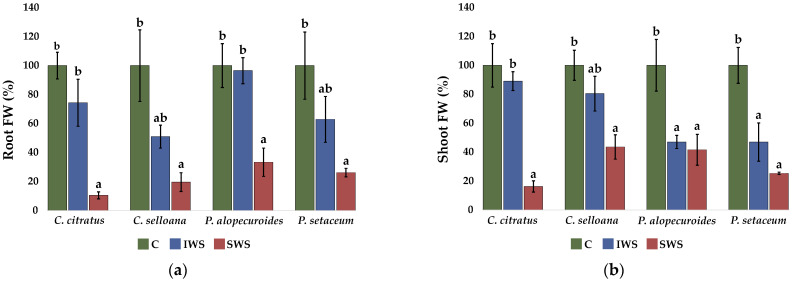

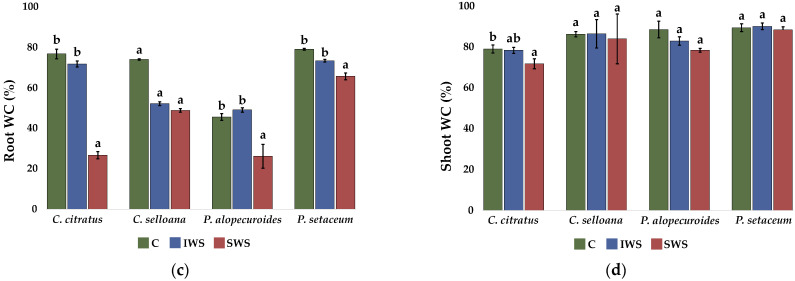
Fresh weight (FW) and water content (WC) of roots (**a**,**c**) and shoots (**b**,**d**) of plants after 30 days of stress treatments. Values are means ± SE (*n* = 5). For each studied species, root and shoot FW of stressed plants are expressed as percentages of the average control values for each parameter, taken as 100%. Absolute control values for root FW are: 4.73 g, 1.09 g, 1.03 g and 4.13 g, for *C. citratus*, *C. selloana*, *P. alopecuroides* and *P. setaceum*, respectively; the corresponding values for shoot FW are: 6.25 g, 4.51 g, 3.50 g and 15.20 g, respectively. Different lowercase letters over the bars indicate significant differences between treatments (CON—control; IWS—intermediate water stress; SWS—severe water stress), for each species, according to the Tukey test (*p* < 0.05).

Young plants of the four investigated species were subjected to intermediate (IWS) and severe (SWS) water stress treatments for 30 days. Vegetative growth was negatively affected by water deficit stress in all species, although with quantitative differences between taxa, as established by the determination of different growth parameters, such as fresh weight (FW) and water content (WC) of roots and shoots, or dry weight (DW) of roots, shoots, and whole plants (Figure 6, Table 3).

There were large variations between the four species in the fresh weight of the control plants. For example, the mean FW of the *P. setaceum* aerial component is around 4-fold and 2.4-fold higher than those of *P. alopecuroides* and *C. citratus*, respectively. For a more precise assessment of the impacts of water deficiency on the plants, all FW data were reported as percentages of the respective controls, which were taken as 100% (Figure 6a,b. The water stress treatments caused a relative reduction in the FW of roots and shoots in plants of the four analysed species. Mean root FW values decreased in response to increasing water stress intensity, but for all species, the differences with the controls were significant only under severe stress (SWS) (Figure 6a). This reduction in FW was partly due to the stress-induced dehydration of the roots since root WC also decreased significantly in the SWS treatment, except for *P. alopecuroides* (Figure 6c). Similar to the effect on roots, mean shoot FW also decreased under stress, although in this case, in plants of the two *Pennisetum* species, the reductions with respect to the corresponding controls were significant not only under severe stress but also in the IWS treatment. Under SWS, shoot FW was reduced to 16%, 44%, 42% and 25% of the corresponding controls in *C. citratus*, *C. selloana*, *P. alopecuroides* and *P. setaceum*, respectively (Figure 6b). In all four taxa, plant shoots appeared to be quite resistant to dehydration under our experimental conditions; a significant, albeit slight decrease in shoot WC was only detected in *C. citratus* plants under severe water stress (Figure 6d). Therefore, the observed reduction in shoot FW was due exclusively to growth inhibition; that is, a relatively lower biomass accumulation in the stressed plants compared to the non-stressed controls. Considering this variable, *P. alopecuroides* seemed the most tolerant to SWS, followed by *C. selloana* and *P. setaceum*, with *C. citratus* being the most sensitive; however, the two *Pennisetum* species were relatively less resistant under IWS conditions.

This water stress tolerance ranking was confirmed considering variations of DW of shoots or whole plants in response to severe water stress. For example, growth inhibition, in terms of whole plant DW reduction with respect to the non-stressed controls, amounted to 36% in *P. alopecuroides*, 50% in *C. selloana*, 70% in *P. setaceum* and 76% in *C. citratus* plants (Table 3).

### 2.4. Biochemical Analyses

Photosynthetic pigments, namely, chlorophylls *a* and *b*, and total carotenoids, were quantified spectrophotometrically in all harvested plants. Overall, pigment contents did not vary much in response to the water stress treatments. The mean values of the three pigments showed a general decreasing trend in plants of *C. citratus*, *C. selloana* and *P. alopecuroides* subjected to the water stress treatments, especially under severe water stress (SWS); on the contrary, a slight increase was observed in *P. setaceum*. However, the differences with the control, non-stressed plants were, in most cases, statistically non-significant. For example, only *P. alopecuroides* showed a significant decrease in all three pigments from their control levels in the intermediate water stress (IWS) treatment (Table 4).

Shoot contents of common plant osmolytes, namely, proline (Pro) and total soluble sugars (TSS), were also determined in all plant samples. Pro levels did not vary significantly in *C. selloana* or *P. alopecuroides* in response to the stress treatments. On the other hand, *C. citratus* and *P. setaceum* registered a significant increase in Pro contents, of 2.3- and 3.6-fold, respectively, but only under SWS conditions. In any case, absolute Pro concentrations remained very low, below 10 µmol g^−1^ DW, and could not have any substantial osmotic effect.

Similarly, no significant changes in TSS content were observed in any of the investigated species in the IWS treatment, or in *C. citratus* and *P. alopecuroides* under severe water deficit (SWS); however, TSS levels increased in *C. citratus* and *P. setaceum* by 1.5- and 2.8-fold, respectively, in the SWS treatment (Table 4). It must be noted that, in non-stressed plants, TSS contents in *C. selloana* and *C. citratus* were about 3-fold higher than in *P. alopecuroides* and more than 5-fold higher than in *P. setaceum* (Table 4).

Lastly, antioxidant metabolites such as total phenolic compounds (TPC) and the subgroup of flavonoids (TF) were also quantified in leaf samples of all harvested plants. No significant differences between control and stressed plants were observed, regardless of the species or the water stress intensity (Table 4).

The application of water stress did not significantly modify the shoot Na^+^ and K^+^ contents (Table 5). However, some slight (but statistically significant) changes were observed in the concentrations of these cations in roots. Under severe stress (SWS), mean root Na^+^ contents were lower than in the control or in the IWS treatment in *C. citratus*, whereas they were higher in *P. setaceum* and did not vary in the other two species. On the other hand, root K^+^ concentrations were not affected by the water deficit treatments in *C. citratus* and *C. selloana* plants but increased in *P. alopecuroides* when comparing stressed and non-stressed plants, and also in *P. setaceum*, although in this case the only significant differences were observed between the IWS and SWS treatments (Table 5). It should be mentioned that in the four species and for each specific treatment, the concentrations of both cations were always higher in shoots than in roots, with differences generally ranging between 1.5- and 3-fold (Table 5).

### 2.5. Multivariate Analysis

**Figure 7:**
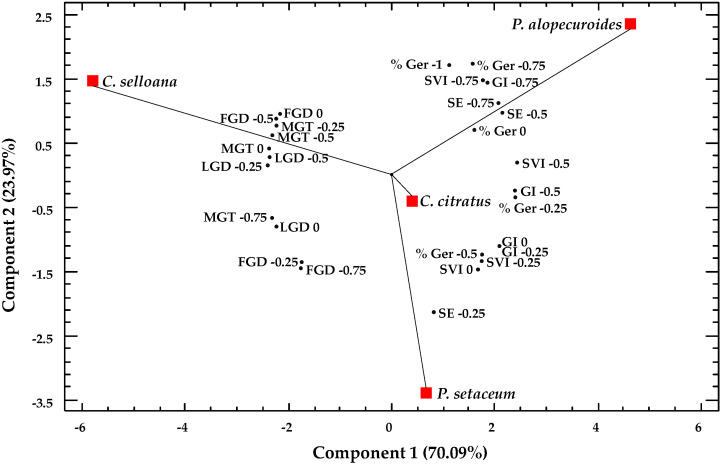
Principal Component Analysis of germination data. Loading and scatter plots of the PCA scores were conducted with germination and seedling’s traits of the species *C. citratus*, *C. selloana*, *P. alopecuroides* and *P. setaceum*. Abbreviations: % Ger, final percentage of germination; MGT, mean germination time; FGD, first germination day; LGD, last germination day; SE, speed of emergence; GI, germination index; SVI, seedling vigour index.

A Principal Component Analysis (PCA) was performed with mean values of germination and seedlings data (Figure 7). The PCA grouped the considered variables and reduced them to two new variables or principal components. These two components had an eigenvalue higher than one, summing up to 94% of the total variance, corresponding 70.09% to the first component and 23.97% to the second component. This graphic representation of the loadings and scores in components 1 and 2 showed the distribution of the studied species according to the considered variables. To simplify the graphic, only the variables with higher weight were represented.

The first component explained most of the total variability of the included data. The variables with the highest weight value in this component were those related to germination percentage, seed vigour and the germination index at high osmotic potential, all positively correlated. Variables with a negative correlation were those related to germination velocity. Based on these results, *C. selloana* showed a considerable distance from the other three species, mainly due to its low germination velocity. *P. alopecuroides* was situated at the other extreme of the graph, with the best scores regarding germination percentage and velocity, even at the lowest osmotic potentials; *C. citratus* and *P. setaceum* showed an intermediate response.

With respect to the second component, *C. citratus* and *P. setaceum* appeared separated due to the differences in germination percentages and velocity at the lowest osmotic potentials. *P. alopecuroides* and *C. selloana* were in the opposite position with respect to *P. setaceum*, according to their higher germination response, particularly at the lowest osmotic potentials. The PCA clearly indicated that *P. alopecuroides* was the species with the best seed germination responses to osmotic stress.

**Figure 8:**
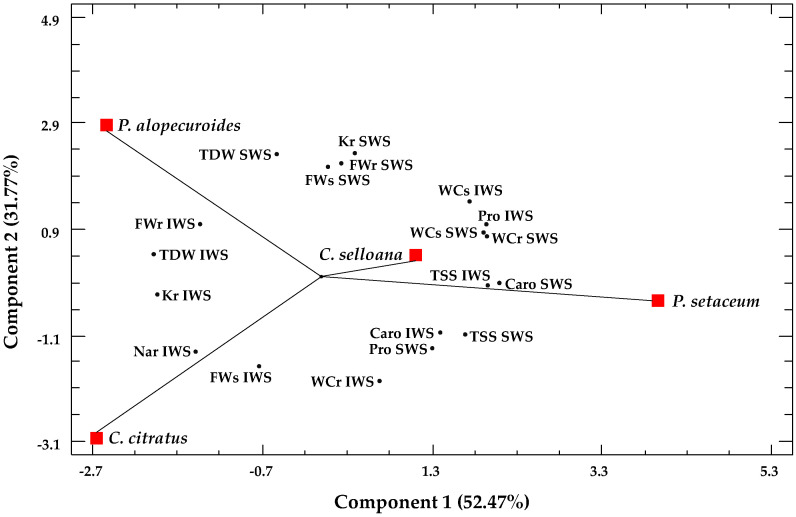
Principal Component Analysis of growth and biochemical data of *C. citratus*, *C. selloana*, *P. alopecuroides* and *P. setaceum*. Loading and scatter plots of the PCA scores were conducted only with the parameters that showed a significant correlation. Abbreviations: IWS, intermediate water stress; SWS, severe water stress; TDW, total dry weight; FWr, root fresh weight root; FWs, shoot fresh weight; WCr, root water content; WCs, shoot water content; Caro, carotenoids; Pro, proline; TSS, total soluble sugars; Kr, root potassium content; Nar, root sodium content.

A second principal component analysis combined the growth and biochemical parameters (Figure 8). Only those variables showing significant changes were considered. Three components with an eigenvalue higher than one were found, covering 100% of the variability of data. The first component, explaining 52.5% of the variation, was positively correlated with the water content of shoots and roots, and with carotenoids, proline and TSS concentrations, and negatively correlated with K^+^ and Na^+^ contents in roots. The second axis explained an additional 31.7%, and it was positively correlated with the root fresh and dry weights and K^+^ contents in plants from the SWS treatment and negatively correlated with the shoot fresh weight of plants from the IWS treatment. The four species were clearly separated according to the two components. The scatterplot of *C. citratus* on the lower part of the graph and *P. alopecuroides* on its upper part was outstanding, indicating a different response under the severe water stress treatment: the first species was the most tolerant to water stress and the second the most susceptible of the four analysed.

## 3. Discussion

Successful germination is critical for the effective propagation and occupation of ecological niches by seed-producing plants and for expanding their distribution range [51,52]. Similarly, efficient germination and seed dispersal, together with vegetative propagation, are decisive factors in increasing the invasive potential of alien species [52,53,54]. The profuse production of seeds with high vigour and germination success is distinctive of these species; these features favour their initial establishment in unoccupied niches and reduce interspecific competition [50,52].

Invasive species’ ability to germinate under a wide range of ecological niches has been attributed to their tolerance to a broad spectrum of adverse environmental conditions [19,55]. Reports documenting such germination potential are continuously increasing as part of the risk assessment necessary for their management [56,57].

Arid environments are particularly vulnerable and potentially affected by colonisation of alien and invasive species, even more so considering the current climate change scenario. The decrease in water potential causes osmotic stress that negatively affects seed germination. In this sense, regarding germination under osmotic stress mimicking drought conditions, the four invasive grass species studied in this work had relatively high germination success. The degree of this effect varies depending on the tolerance of specific species and is usually related to adaptation to stressful habitats such as deserts or salt marshes.

The best invasive capacity of the four analysed species was observed in the two *Pennisetum* taxa, as shown by their highest germination velocity and seedlings’ vigour index. The PCA of germination responses points to *P. alopecuroides* as the species with the maximum invasive potential in dry environments. In agreement with previous reports from China [58], *P. aloperucoides* seeds showed more successful and rapid germination in all treatments than that recorded in *P. setaceum*.

In the germination assays, *C. citratus* was the species most affected by osmotic stress, as its seeds did not germinate at all when the highest PEG concentration was applied. However, its response was comparable to the other studied species under milder stress conditions, at least up to −0.75 MPa. On the other hand, *P. alopecuroides*—only recently recognised as invasive in some areas in the USA [26]—was the least impacted, with half of its seeds retaining their germination capacity under an osmotic potential of −1 MPa. Meanwhile, although *P. setaceum* has been included on the “List of invasive alien species of Union concern”, according to the EU regulation 1143/2014 [59], our findings suggest that it may be less potent in its invasiveness of arid areas due to its reduced germination and seedling vigour under the applied drought conditions during germination and seedling elongation. These data agree with previous studies [60], whereby dry summers may detrimentally affect its ability to produce seeds and successful seedling establishment [61].

As for *C. selloana*, its germination success decreased gradually in parallel to increasing PEG concentrations, although it maintained some germination capacity even at the lowest osmotic potential tested. Of the analysed species, the pampas grass had the longest germination time in control conditions and under stress. Previous studies ranked this species as one of the worst invaders in Europe [48,62,63]. Indeed, its potency as an invasive and widespread species is supported by several traits, such as the large production of viable small seeds, which are easily dispersed by wind at long distances, the capacity to germinate in a wide range of environments and substrates [64,65], the efficient use of limited resources and its rapid adaptability to changes in resource availability [66,67]. These authors also verified that the seeds remain viable for several weeks under water stress conditions, suggesting a staggered germination strategy that allows it to overcome unfavourable periods, delaying the germination of a part of the seeds.

Exposure to sufficiently low water potentials in the substrate, which prevents germination, can alter seeds’ germination responses when stressful conditions are alleviated. Seeds of some species show a priming effect after salt or osmotic stress treatments, and several studies reported a significant effect on germination percentage, mean germination time or seed vigour in seeds exposed to low water potentials [68,69,70]. Previous exposure to stress sometimes leads to earlier and increased germination due to enzyme activation, increased levels of germination-promoting metabolites, and osmotic adjustment [71]. This osmopriming effect was not observed in any of the four species studied here, as none of the applied treatments induced a significant increase in the percentage or speed of germination. As with other Poaceae, the seeds of the four species exposed to water restrictions maintained their viability and resilience to desiccation, showing germination percentages and rates similar to those in controls [72]. Soil moisture in areas with Mediterranean climate is characterised by strong seasonal variation with a reduction in their water potential to less than −2 MPa in summer [73], when maximum drought is experienced. There is also a large spatial heterogeneity, with stronger fluctuations at smaller depths. At 0.3 m depth, the soil water potential in a Mediterranean oak forest decreased to −2.3 at the end of summer but returned quickly to 0 values in autumn after rainfall events [73]. Due to their recovery capacity, seeds of the four grasses have the possibility to take advantages of the alleviation of the soil water scarcity after rainfalls, as many other Mediterranean species. However, a key factor in increasing their competitivity is their ability to maintain high percentages of germination under lower water potential in soil.

The hydrotime model describes the germination response to osmotic stress and its relationship with the adaptive responses of species to their habitats [74]. θH is related to the period between the start of imbibition and the start of radicle emergence and, consequently, the time required for germination, whereas Ψb measures the range of water potentials in which seeds can germinate [74]. The studied species showed hydrotime values from 2.3 to 1.8 MPa·days. These values are low if compared to different populations of the Poaceae *Festuca pallescens* from Patagonia, with values ranging from 30.9 to 13.6 MPa·days [74], or different *Stipa* species of cool and warm habitats, with values between 31 and 173 MPa·days [75]. Compared to other species of the same family, the four grasses examined in this study require shorter time for seed germination, which gives them a competitive advantage over native species.

One of the germination parameters most used in the literature is the base water potential for a 50% inhibition of the maximum germination (Ψb_50_). When comparing the studied species with non-invasive plants, the obtained Ψb_50_ values are generally lower [72,74], confirming their higher ability to germinate in a wide range of osmotic potentials. These data reveal the hydrotime model as a valuable tool for evaluating the invasive capacity of alien species.

The work reported here mainly focuses on analysing seed germination responses to osmotic stress, as germination is, in general, the plant developmental phase more sensitive to stress and a critical step in invasion processes [52]. However, the responses of young plants of the studied species to drought and its detrimental impacts on vegetative growth were also documented. Here, the expected trade-off between vigorous growth under optimal conditions and reduced tolerance to stress [76] was evident. For instance, *P. setaceum* and *C. citratus* plants, which showed the highest relative biomass accumulation under control conditions, experienced the strongest relative growth reduction when stress was applied. Under stress, plants divert resources from biomass accumulation to activating defence mechanisms, such as osmotic adjustment, which is ensured through the build-up of osmolytes such as proline and soluble sugars [77]. These findings partly explain the relative geographical and environmental distribution of the studied panel of species. Lemongrass, for example, is a species of tropical and sub-tropical character, cultivated mainly in Asia, South and Central America, and Africa as a culinary and medicinal herb [78], and has been reported as invasive exclusively in areas with similar climatic conditions [35]. Recently, *C. citratus* has been introduced in Spain in an area near the Albufera Lake in Valencia; our study suggests that this species will not become problematic for this region as the climate is generally too dry for its potential spread. As for the fountain grass (*P. setaceum*), its documented fast and vigorous growth [50,79], which contributes to its invasiveness potential, will probably cause the rapid consumption and early depletion of the available water, inducing, in turn, the reduction in its growth under stress. This feature is typical of plants with a large leaf surface area and high evapotranspiration rates [80].

Another morphological trait that may be relevant for the invasive potential of a species is the alteration of biomass allocation patterns in favour of roots. The two taxa showing an increase in the root/shoot FW ratio under moderate drought were *P. setaceum* and *P. alopecuroides*, which may explain why *P. setaceum* is an excellent invader of habitats characterised by fluctuating resources, frequently disturbed or with an irregular rainfall pattern [60]. However, as commented previously, soil moisture may limit the dispersion of *P. setaceum* during germination and early growth stages but not that of the more stress-tolerant *P. alopecuroides*. Regarding *C. selloana*, it has been reported as tolerant to drought and warm temperatures [81], and its expansion is clearly favoured by environmental disturbances [82], bare ground, low pH and low functional group richness [83]. Its high phenotypic plasticity in response to variations in N availability and water table depth, typical for coastal and riparian environments, favour the presence of this species in wetlands, which are fragile ecosystems, reducing the natural biodiversity of such areas [63,84].

Photosynthetic pigment contents did not vary substantially in response to water stress in any of the analysed species; only small fluctuations, in most cases statistically non-significant, were observed when comparing the different treatments. On the other hand, Pro and TSS contents increased significantly only in the leaves of *C. citratus* and *P. setaceum* plants subjected to severe stress, which would suggest that both osmolytes are involved in the response to water deficit stress in these two species. Proline represents one of the most common osmolytes in plants. Besides its direct function in osmotic adjustment under stress, Pro plays other major roles in the responses to stress, as a low-molecular-weight chaperon, a metal chelator, an ROS scavenger involved in antioxidant defence reactions, or a signalling molecule [85,86]. In the four analysed taxa, even the maximum absolute Pro concentrations reached are too low to have a relevant osmotic effect. However, considering its additional biological functions, which may not require such high concentrations, Pro participation in drought tolerance mechanisms in these species cannot be ruled out. TSS are also involved in many physiological processes, such as photosynthesis, seed germination, flowering, or senescence, apart from their important role in osmotic regulation; therefore, changes in TSS concentrations should be interpreted with care as they may not be related to stress defence mechanisms [87,88].

Regarding the monovalent cations analysed, no significant stress-induced changes in Na^+^ or K^+^ concentrations have been detected, in either roots or shoots, except for an increase in K^+^ in *P. alopecuroides* roots. This probably contributes to drought tolerance in this species, the most tolerant of the four studied according to the relative inhibition of growth under SWS conditions. Nutrient acquisition by roots is usually reduced under drought conditions, which restricts K^+^ diffusion in the soil towards the absorption zone of the roots [89]. Potassium is a key element for multiple physiological processes in plants, and its homeostasis is a general adaptive trait to different environmental stresses [90,91]. For example, a better K^+^ retention in roots, resulting from the stress-induced activation of the H^+^-ATPase, was one of the mechanisms explaining the higher salt tolerance of *Brassica napus* compared to two other congeners [92]. Potassium plays essential functions in plants’ responses to drought due to its roles in maintaining cell turgor, osmotic adjustment, aquaporin regulation and hydraulic conductance of the xylem. A close relationship between the K^+^ nutritional status and plant drought resistance has been demonstrated [91]. It should also be highlighted that Na^+^ and K^+^ concentrations were consistently higher in shoots than in roots in the four species and all treatments. This points to mechanisms of active transport of both cations from roots to the aerial part of the plants, where the ions will contribute to osmotic adjustment as ‘inorganic osmolytes’ and, thus, to drought tolerance.

## 4. Materials and Methods

### 4.1. Plant Material

Four ornamental grass species (Figure 1), some with very high invasiveness according to the ‘Invasive Species Compendium’, were used in the presented study. For the experimental setup, seeds procured from commercial suppliers (CANTUESO Natural Seeds, Córdoba, Spain), collected from the wild in autumn 2021, or from germplasms banks (Botanical Garden of Valencia, Valencia, Spain), were utilised as starting material (seed’s sources and general species overview are detailed in Table 6). Seed germination and vegetative plant growth before and during the experimental treatments were performed in the laboratories and greenhouses of the Institute for the Conservation and Improvement of Valencian Agrodiversity (COMAV), Polytechnic University of Valencia, Valencia, Spain.

### 4.2. Germination Assays

Collected seeds (*C. selloana* and *P. setaceum*) were sterilised and stored in paper bags in a dry, controlled environment at a temperature of 20–22 °C and 40–50% relative humidity (RH) for ten days. In vitro germination assays were performed using four replications per treatment and species, each containing 25 seeds placed in 55 mm-diameter Petri dishes on a double layer of filter paper. The paper was moistened with 1.5 mL of distilled water for the control or with increasing concentrations of PEG 6000 (Polyethylene Glycol), generating osmotic potentials of −0.25 MPa, −0.5 MPa, −0.75 MPa and −1 MPa. PEG amounts required to provide the indicated osmotic potentials were calculated by applying the Van’t Hoff equation [93]. The Petri dishes were incubated in a growth chamber at 25 °C under a 12 h photoperiod inside transparent zipped bags to avoid evaporation.

The number of germinated seeds, identified as those with a minimum of 1 mm radicle emergence, was registered daily over 30 days. The germination capacity was expressed as the percentage of germination (GP) and the germination rate as mean germination time (MGT), which was calculated according to the formula:

MGT = ∑ Dn/∑n(1)

where D represents the number of days from the beginning of the germination test, and n is the number of seeds newly germinated on day D [94].

Moreover, to better compare the response of the different species regarding the osmotic stress-induced inhibition of germination, the “reduction in germination percentage” (RGP) was calculated as indicated [95]:

RGP = [1 − (N° of germinated seeds in PEG/N° of germinated seeds in control)] × 100.(2)

After ten days of germination, the length of radicles and hypocotyls of germinated seeds were measured and analysed using Digimizer v.4.6.1 software (MedCalc Software, Ostend, Belgium, 2005–2016). The following additional indexes were then determined:

Germination index (GI), which is a strong indicator of the success and speed of germination [96], was calculated using the equation:

GI = ∑G/T(3)

where G is the number of germinated seeds on a specific day, and T is the number of days from the start of the experiment till that day.

Speed of emergence (SE) [97], to determine germinative energy through germination speed, calculated using the equation:

SE = [(number of germinated seeds on the first day of germination)/(number of germinated seeds on the last day of germination)] × 100(4)

Seedling vigour index (SVI), using the equation described in [98]:

SVI = (Seedling length, in mm × Germination percentage)/100(5)

The seeds that did not germinate after 30 days in the presence of PEG were included in recovery treatments. The seeds were rinsed, transferred to new Petri dishes with distilled water, and incubated for 20 days under the same conditions as in the previous germination assays. Germination percentages in the recovery assays were calculated following Zaman et al. [99]. The relationship between seed germination rates and water potential in the germination and recovery assays was reported by hydrotime analysis as:

θH = [Ψ − Ψb(g)]tg(6)

where Ψ is the water potential of the external medium (PEG solution); Ψb is the base water potential (the lowest water potential allowing the seeds to germinate), Ψb(g) refers to a specific percentage of germinated seeds, g; and finally, tg is the time needed for germination of that fraction of seeds, g% [100,101], extrapolated as regression lines. The Hydrotime (θ) was calculated as the inverse of the slope of the corresponding regression lines [102]. The base water potential, Ψb, was calculated for all the species, and the base water potential was also calculated for 50% inhibition of the maximum germination (Ψb_50_).

### 4.3. Plant Growth and Water Stress Treatments

Seedlings from the controls in the germination assays mentioned above were transplanted manually into plastic pots (12 cm diameter) filled with commercial peat (26% organic carbon, pH = 7.0, and EC = 0.6 dS m^−1^), placed in the greenhouse and watered manually twice a week with tap water. During the experiments, the following conditions were recorded: temperatures between 22.1 ± 1.6 and 29.3 ± 2.1 °C, and RH between 65.7 ± 8.7% and 93.2 ± 3.1% under a photoperiod of 16 h light.

Five weeks after transplanting the seedlings, when the plantlets were fully developed, the water stress treatments were initiated, using five biological replicas (individual plants) per species and treatment. The pots were placed in plastic trays (10 pots per tray) and watered twice per week with tap water added to each tray. Control plants received 1.5 L in each irrigation, plants were subjected to the intermediate water stress treatment (IWS) with half of this amount (0.75 L), and those of the severe water stress treatment (SWS) were not watered at all. After four weeks, when the soil moisture of the SWS plants reached 5–8%, the plants were harvested and processed for further biochemical analysis. The aerial parts were separated from the roots, the latter were cleaned with a brush, and both parts (roots and shoots) were measured separately.

The fresh weight of roots and shoots was determined for all individual plants (five per treatment and species). A fraction of the harvested material was weighed before (fresh weight, FW) and after drying at 65 °C for 72 h (dry weight, DW), and the water content of roots and shoots was calculated using the following equation:

WC% = [(FW − DW)/FW] × 100(7)

Fresh plant material was frozen in liquid N_2_ and stored at −75 °C, and dry material was kept at room temperature in tightly closed tubes.

### 4.4. Photosynthetic Pigments

Fresh shoot material (50 mg) was ground and extracted overnight in ice-cold 80% acetone. The absorbance of the supernatant was then measured spectrophotometrically at 470 nm, 646 nm and 663 nm. Chlorophyll *a* (Chl a), chlorophyll *b* (Chl b) and carotenoids (Caro) contents were calculated in mg g^−1^ DW, applying the equations detailed in Lichtenthaler and Wellburn [103].

### 4.5. Ion Content Measurements

The concentrations of sodium (Na^+^) and potassium (K^+^) were calculated separately for roots and shoots, following the protocol described by Weimberg [104]. About 100 mg of ground dry material was extracted in boiling Milli-Q water, cooled on ice and filtered through a 0.45 µm Gelman nylon filter (Pall Corporation, Port Washington, NY, USA). The extracts were then appropriately diluted before cation measurements with a 410C flame photometer (Sherwood Scientific Ltd., Cambridge, UK).

### 4.6. Quantification of Osmolytes

Shoot proline (Pro) contents were quantified as previously described [105]. Fresh ground material (50 mg) was extracted in 3% (*w*/*v*) aqueous sulphosalicylic acid; the samples were mixed with acid ninhydrin, incubated in a water bath for 1 h at 95 °C, cooled on ice, and then extracted with toluene. The absorbance of the organic phase was measured spectrophotometrically at 520 nm, using toluene as the blank. Samples of known Pro concentration were assayed in parallel, to obtain a standard curve, and Pro concentrations were expressed as µmol g^−1^ DW.

Total soluble sugars (TSS) were determined following the method of Dubois et al. [106]. Fresh ground material (50 mg) was extracted overnight with 80% (*v*/*v*) methanol. After centrifugation, 5% phenol and concentrated sulphuric acid were added to the supernatant to induce an exothermic reaction to caramelise the extracted sugar contents. Spectrophotometric measurements were then performed at 490 nm. TSS concentrations were expressed as equivalents of glucose, used as the standard (mg eq. glucose g^−1^ DW).

### 4.7. Determination of Antioxidant Compounds

Total phenolic compounds (TPC) and total flavonoids (TF) were quantified in the same methanol extracts used for measuring total soluble sugars (TSS). TPC were determined by the method of Blainski et al. [107], by reaction with the Folin–Ciocalteu reagent and Na_2_CO_3_; the reaction mixtures were incubated at room temperature for 90 min, in the dark, and the absorbance measured at 765 nm. Gallic acid (GA) was used as the standard, and TPC concentrations were expressed as equivalents of GA (mg eq. GA g^−1^ DW). Total flavonoids (TF) were quantified following the protocol of Zhishen et al. [108], based on the nitration with NaNO_2_ of aromatic rings containing a catechol group, followed by a reaction with AlCl_3_ at basic pH. After the reaction, the absorbance of the samples was measured at 510 nm, and TF contents were expressed as equivalents of catechin, used as the standard (mg eq. C g^−1^ DW).

### 4.8. Statistical Analysis

The statistical analyses of the data were performed using SPSS Statistics statistical software (IBM SPSS Statistics) and Statgraphics Centurion XVI (Statgraphics Technologies, The Plains, VA, USA).

Analysis of variance (one-way ANOVA) was used to estimate the effects of the stress treatments on the traits analysed for each species. The Tukey test was used as a post hoc test, at *p*-value of 0.05 (*p* < 0.05), to analyse the differences if the null hypothesis was rejected. Principal Component Analyses were performed separately for germination and plant growth, considering the mean values of significant growth and biochemical parameters determined for plants of the four investigated species subjected to the different treatments.

## 5. Conclusions

Our study indicates that special attention should be given to *P. alopecuroides* because of its high invasive potential, which is not generally recognised. The species is now widely commercialised in Europe as ornamental and preferred to other *Pennisetum* species that are catalogued as invasive. However, its ability to tolerate water stress well, both at the seed germination stage and during vegetative growth, will favour the spread of this species in areas with a dry climate such as the Mediterranean. One distinctive characteristic of this species, contributing to its higher drought tolerance, appears to be the increase in root K^+^ concentration in response to drought conditions. On the contrary, the possibility of *C. citratus* becoming invasive in such environments is very low, as its seed germination capacity and plant vegetative growth proved to be the most susceptible to drought of the four analysed species. The other two species under study, *C. selloana* and *P. setaceum*, are catalogued as aggressive invaders in many areas of the world. These plants showed a moderate tolerance to drought during vegetative growth and maintained their germination ability even under the lowest osmotic potential applied. Therefore, they may represent a threat to many ecosystems in the Mediterranean, especially considering that climate change is progressively increasing drought conditions in this region.

## Figures and Tables

**Table 1:** Base osmotic potential for maximum germination (Ψb), base water potential for a 50% inhibition of the maximum germination (Ψb_50_) and hydrotime (θ), measured for each studied species.

Parameter	*C. citratus*	*C. selloana*	*P. alopecuroides*	*P. setaceum*
Ψb (MPa)	−1.0	−0.7	−1.3	−1.0
Ψb_50_ (MPa)	−1.4	−1.1	−2.6	−1.2
θ (MPa·days)	2.1	2.3	1.9	1.8

**Table 2:** Seedling’s parameters (means ± SE; *n* = 4), calculated for the four studied species in the 30-day germination assays. Different lowercase letters indicate significant differences between treatments per species, according to the Tukey test (*p* < 0.05). n.g.: no germination.

Parameter	Treatment (MPa)	*C. citratus*	*C. selloana*	*P. alopecuroides*	*P. setaceum*
Radicle length (mm)	Control	19.3 ± 0.4 d	4.4 ± 0.3 ab	23.9 ± 0.5 d	19.7 ± 2.7 c
	−0.25	15.6 ± 0.2 b	5.7 ± 0.3 b	20.6 ± 0.4 c	17.2 ± 0.7 c
	−0.5	18.3 ± 0.2 c	4.3 ± 0.2 a	17.9 ± 0.1 b	9.9 ± 0.2 b
	−0.75	12.6 ± 0.2 a	3.8 ± 0.3 a	17.4 ± 0.0 b	3.9 ± 0.1 a
	–1.0	n.g.	3.5 ± 0.3 a	3.7 ± 0.0 a	0.9 ± 0.5 a
Radicle length reduction (%)	−0.25	18.8 ± 1.2 a	–28.1 ± 7.7 a	14.2 ± 1.8 b	13.0 ± 3.6 a
	−0.5	5.2 ± 1.1 b	3.4 ± 3.6 b	25.1 ± 0.2 b	49.7 ± 1.1 b
	−0.75	34.8 ± 0.9 c	12.9 ± 6.5 b	27.4 ± 0.1 c	80.3 ± 0.7 bc
	−1.0	n.g.	19.8 ± 7.3 c	84.4 ± 0.2 d	95.6 ± 2.5 c
Hypocotyl length (mm)	Control	18.2 ± 0.4 c	10.4 ± 0.4 c	24.5 ± 0.3 e	36.1 ± 1.4 c
	−0.25	15.3 ± 0.2 b	10.0 ± 0.3 c	20.9 ± 0.1d	29.2 ± 0.5 c
	−0.5	14.2 ± 0.1 b	4.9 ± 0.4 b	19.8 ± 0.3 c	13.9 ± 0.3 b
	−0.75	10.8 ± 0.3 a	3.9 ± 0.7 ab	11.0 ± 0.1 b	2.4 ± 0.2 a
	−1.0	n.g.	1.3 ± 0.2 a	2.3 ± 0.03 a	0.7 ± 0.5 a
Hypocotyl length reduction (%)	−0.25	15.7 ± 0.9 b	3.8 ± 3.3 a	14.6 ± 0.6 b	19.2 ± 1.3 b
	−0.5	22.0 ± 0.6 c	52.2 ± 3.9 b	19.1 ± 1.2 b	61.3 ± 0.9 c
	−0.75	40.3 ± 1.5 d	62.3 ± 6.4 b	55.1 ± 0.4 c	93.3 ± 0.6 d
	−1.0	n.g.	86.6 ± 1.7 c	90.3 ± 0.1 d	97.9 ± 1.3 d
Seedling vigour index	Control	17.3 ± 0.4 c	8.9 ± 0.7 d	23 ± 0.5 e	31.5 ± 2.7 c
	−0.25	13.3 ± 0.1 b	7.1 ± 0.3 c	19.9 ± 0.5 d	25.9 ± 0.4 c
	−0.5	11.9 ± 0.4 b	3 ± 0.3 b	19.1 ± 0.4 c	11.4 ± 0.6 b
	−0.75	5.3 ± 0.7 a	1.4 ± 0.3 ab	9.6 ± 0.3 b	0.6 ± 0.1 a
	−1.0	n.g.	0.2 ± 0.0 a	1.1 ±0.1 a	0.1 ± 0.0 a

**Table 3:** Effect of stress treatments on the dry weight of roots and shoots (*n* = 5). Different lowercase letters indicate significant differences between treatments with each species per parameter according to the Tukey test (*p* < 0.05).

Parameter	Treat.	*C. citratus*	*C. selloana*	*P. alopecuroides*	*P. setaceum*
Dry weight roots (g)	CON	1.1 ± 0.1 b	0.3 ± 0.0 a	0.6 ± 0.1 b	0.8 ± 0.2 b
	IWS	1.0 ± 0.2 b	0.2 ± 0.0 ab	0.5 ± 0.1 b	0.7 ± 0.1 ab
	SWS	0.4 ± 0.0 a	0.1 ± 0.0 a	0.3 ± 0.1 a	0.3 ± 0.0 a
Dry weight shoots (g)	CON	2.3 ± 0.3 b	0.8 ± 0.1 a	0.5 ± 0.1 a	2.1 ± 0.3 b
	IWS	2.0 ± 0.3 b	0.6 ± 0.1 ab	0.4 ± 0.0 a	1.0 ± 0.3 a
	SWS	0.4 ± 0.1 a	0.4 ± 0.1 a	0.4 ± 0.1 a	0.6 ± 0.1 a
Total dry weight (g)	CON	3.4 ± 0.5 b	1.0 ± 0.1 b	1.1 ± 0.1 a	2.9 ± 0.5 b
	IWS	3.0 ± 0.5 b	0.8 ± 0.1 b	0.9 ± 0.1 a	1.7 ± 0.4 b
	SWS	0.8 ± 0.1 a	0.5 ± 0.1 a	0.7 ± 0.2 a	0.9 ± 0.1 a

Note: CON—control; IWS—intermediate water stress; SWS—severe water stress.

**Table 4:** Effect of stress treatments on the shoot contents of photosynthetic pigments, osmolytes and antioxidant compounds: chlorophylls *a* and *b* (Chl a and Chl b), carotenoids (Caro), proline (Pro), total soluble sugars (TSS), total phenolic compounds (TPC) and total flavonoids (TF). Values shown are means ± SE (*n* = 5). Different lowercase letters indicate significant differences between treatments for each determined variable and species, according to the Tukey test (*p* < 0.05). GA: gallic acid; C: catechin.

Parameter	Treat.	*C. citratus*	*C. selloana*	*P. alopecuroides*	*P. setaceum*
Chl a (mg g^−1^ DW)	CON	7.3 ± 1.5 a	8.4 ± 0.8 a	12.9 ± 1.6 b	6.0 ± 0.7 a
	IWS	7.1 ± 1.8 a	10.6 ± 1.2 a	6.8 ± 0.5 a	6.8 ± 0.8 a
	SWS	4.3 ± 0.8 a	7.1 ± 0.9 a	8.5 ± 1.2 ab	7.5 ± 0.8 a
Chl b (mg g^−1^ DW)	CON	2.1 ± 0.5 a	2.7 ± 0.2 a	3.6 ± 0.4 b	1.5 ± 0.2 a
	IWS	2.0 ± 0.5 a	3.6 ± 0.3 a	1.9 ± 0.1 a	1.6 ± 0.2 a
	SWS	1.1 ± 0.2 a	2.5 ± 0.4 a	2.5 ± 0.4 ab	2.0 ± 0.2 a
Caro (mg g^−1^ DW)	CON	1.6 ± 0.3 a	1.5 ± 0.1 ab	2.4 ± 0.2 b	1.0 ± 0.13 a
	IWS	1.5 ± 0.2 a	2.0 ± 0.2 b	1.3 ± 0.1 a	1.2 ± 0.2 a
	WS	0.9 ± 0.1 a	1.2 ± 0.2 a	1.5 ± 0.2 a	1.3 ± 0.1 a
Pro (µmol g^−1^ DW)	CON	3.1. ± 0.8 a	4.3 ± 1.1 a	1.1 ± 0.3 a	1.6 ± 0.3 a
	IWS	2.2 ± 0.4 a	6.8 ± 0.9 a	1.5 ± 0.2 a	3.8 ± 0.8 ab
	SWS	7.2 ± 1.6 b	5.9 ± 0.7 a	1.2 ± 0.2 a	5.8 ± 0.7 b
TSS (mg eq. glucose g^−1^ DW)	CON	28.9 ± 4.1 a	28.6 ± 6.8 a	9.8 ± 2.0 a	5.6 ± 0.8 a
	IWS	23.6 ± 5.2 a	45.4 ± 3.3 a	6.9 ± 0.9 a	9.3 ± 1.5 ab
	SWS	43.5 ± 3.2 b	35.4 ± 7.7 a	6.9 ± 0.4 a	15.9 ± 4.2 b
TPC (mg eq. GA g^−1^ DW)	CON	11.6 ± 1.1 a	7.9 ± 0.3 a	8.9 ± 1.0 a	4.8 ± 0.5 a
	IWS	9.9 ± 1.8 a	9.4 ± 0.5 a	6.9 ± 0.8 a	5.7 ± 0.9 a
	SWS	13.7 ± 1.3 a	6.9 ± 1.8 a	5.5 ± 1.2 a	6.4 ± 1.8 a
TF (mg eq. C g^−1^ DW)	CON	6.6 ± 0.7 a	5.5 ± 0.6 a	3.7 ± 0.4 a	3.1 ± 0.3 a
	IWS	4.9 ± 0.9 a	6.9 ± 0.7 a	2.6 ± 0.4 a	4.2 ± 0.5 a
	SWS	6.7 ± 0.9 a	4.3 ± 0.8 a	2.5 ± 0.6 a	3.8 ± 1.3 a

Note: CON—control; IWS—intermediate water stress; SWS—severe water stress.

**Table 5:** Effect of the water stress treatments on sodium (Na^+^) and potassium (K^+^) root and shoot contents in the four investigated species. Values are means ± SE (*n* = 5). Different lowercase letters indicate significant differences between treatments, for each species and measured variable, according to the Tukey test (*p* < 0.05).

Parameter	Treat.	*C. citratus*	*C. selloana*	*P. alopecuroides*	*P. setaceum*
K^+^ shoot (µmol g^−1^ DW)	CON	569.1 ± 125.6 a	785.7 ± 67.0 a	975.9 ± 46.8 a	1104.9 ± 79.7 a
	IWS	458.4 ± 67.1 a	882.8 ± 60.1 a	864.1 ± 91.2 a	1290.7 ± 87 a
	SWS	432.4 ± 45.9 a	934.4 ± 34.7 a	792.7 ± 89.4 a	1164.1 ± 30 a
K^+^ root (µmol g^−1^ DW)	CON	252.5 ± 54.3 a	500 ± 25.3 a	316.2 ± 14.6 a	744.5 ± 46.5 ab
	IWS	336.6 ± 65.5 a	448 ± 16.3 a	400.4 ± 19.3 b	670.0 ± 36.9 a
	SWS	210.3 ± 19.6 a	476.9 ± 37.6 a	397.9 ± 15.2 b	826.2 ± 21.1 b
Na^+^ shoot (µmol g^−1^ DW)	CON	273.4 ± 52.7 a	399.4 ± 25.1 a	417.9 ± 31.1 a	561.7 ± 51.9 a
	IWS	216.6 ± 38 a	422.7 ± 27.2 a	526.6 ± 49.8 a	619 ± 48.1 a
	WS	214.0 ± 42.2 a	433.7 ± 12.9 a	524.8 ± 32.6 a	521.9 ± 14.3 a
Na^+^ root (µmol g^−1^ DW)	CON	160.1 ± 30.2 ab	199.3 ± 12.7 a	189.1 ± 10.4 a	315.6 ± 19.3 a
	IWS	211.6 ± 36.4 b	172.5 ± 13.2 a	200.5 ± 24.6 a	303.2 ± 14.7 a
	SWS	81.1 ± 6.5 a	165.2 ± 19.4 a	203.1 ± 16.6 a	418.2 ± 28.0 b

Note: CON—control; IWS—intermediate water stress; SWS—severe water stress.

**Table 6:** Source of the seeds, native distribution, environmental requirements and invasive potential of the tested species [27,44,45].

Plant Species	*Cymbopogon* *citratus*	*Cortaderia* *selloana*	*Pennisetum* *alopecuroides*	*Pennisetum* *setaceum*
**Source of the seeds**	CANTUESO Natural Seeds	The Botanical Garden, Valencia, Spain	CANTUESO Natural Seeds	Collected from the wild, Valencia, Spain
**Native distribution**	Sri Lanka	Asia and Africa	Eastern Africa	South America
**Environmental** **requirements**	Full sun, moist soil	Full sun, sandy soil	Full sun, moist soil	Medium shade
**Invasive potential**	High	High	High	High

## Data Availability

Data are contained within the article and Supplementary Material.

## Supplementary Materials

The following supporting information can be downloaded at: https://www.mdpi.com/article/10.3390/plants12061260/s1, Table S1: Parameters of germination with mean values ± SE of four invasive grass species after 30 days of applied osmotic stress and after 20 days of recovery. Different lowercase letters within the bars indicate significant differences between treatments within each species, according to Tukey post hoc test (*p* < 0.05). Abbreviations: RGP, reduction in germination percentage; FGD, first germination day; LGD, last germination day; TSG, time spread germination; SE, speed of emergence; GI, germination index.
