# Supplementary material for: Effect of Water Deficit on Germination, Growth and Biochemical Responses of Four Potentially Invasive Ornamental Grass Species
Source: Plants (Basel). 2023 Mar 10;12(6):1260. doi: 10.3390/plants12061260 (PMC10053442; doi:10.3390/plants12061260)
Supplement: Supplementary file 1 [file plants-12-01260-s001.zip › plants-2216760-supplementary.pdf]

Table S1. Parameters of germination with mean values ± SE of four invasive grass species after 30 days of applied osmotic stress and after 20 days of recovery treatment after 20 days. Different lowercase letters within the bars indicate significant differences between treatments within one species, according to Tukey post-hoc test ($p < 0.05$). Abbreviations: RGP, reduction germination percentage; FGD, first germination day; LGD, last germination day; TSG, time spread germination; SE, speed of emergence; GI, germination index. n.g., no germination

Parameter	Assay	Treatment	*C. citratus*	*C. selloana*	*P. alopecuroides*	*P. setaceum*
RGP (%)	Germination	-0.25	8.6 ± 1.0 a	17.4 ± 2.2 ab	-1.3 ± 2.2 a	-2.3 ± 2.2 a
		-0.5	11.7 ± 3.0 a	29.1 ± 2.2 bc	17.9 ± 1.1 b	5.7 ± 4.0 a
		-0.75	48.2 ± 7.1 b	53.5 ± 9.9 c	6.7 ± 2.7 ab	69 ± 4.7 b
		-1	100.0 ± 0.0 c	79.1 ± 3.0 d	46.7 ± 4.4 c	95.4 ± 2.7 c
FGD (days)	Germination	C	1.0 ± 0.0 a	2.0 ± 0.0 a	1.0 ± 0.0 a	1.0 ± 0.0 a
		-0.25	2.0 ± 0.0 b	2.0 ± 0.0 a	1.2 ± 0.3 a	2.0 ± 0.0 a
		-0.5	2.7 ± 0.3 c	3.5 ± 0.3 ab	2 b	2.0 ± 0.0 a
		-0.75	3.2 ± 0.3 d	4.5 ± 0.3 b	2 b	4.7 ± 0.5 b
		-1	n.g.	9.5 ± 1.2 c	2.2 ± 0.3 b	8.2 ± 4.8 c
	Recovery	-0.25	-	10.7 ± 3.8	-	-
		-0.5	1.0	2.0	-	1.0
		-0.75	1.0	2.0	1.3 ± 0.3	1.0
		-1	1.0	3.0	1.0	1.0
LGD (days)	Germination	C	5.5 ± 0.9 a	10.5 ± 0.5 a	2.2 ± 0.3 a	8.2 ± 3.7 a
		-0.25	7.2 ± 1.3 a	15.5 ± 1.7 ab	4.2 ± 0.6 a	7.2 ± 1.4 a
		-0.5	13.5 ± 2.5 ab	29.2 ± 0.3 b	8.7 ± 3.1 ab	13.5 ± 4.5 a
		-0.75	22 ± 2.7 b	20 ± 3.5 b	9.2 ± 1.7 ab	14.5 ± 2.6 a
		-1	n.g.	20 ± 1.0 c	12.5 ± 1.6 b	8.7 ± 5.1 a
	Recovery	-0.25	-	14.7 ± 2.8	-	-
		-0.5	3.0 ± 2.0	6.7 ±2.6	-	1.7 ± 0.3
		-0.75	1.7 ± 0.3	9.7 ± 1.3	1.3 ± 0.3	5.7 ± 2.9
		-1	3.3 ± 0.3	8 ± 1.7	3.8 ± 1.1	7 ± 2.1
TSG (days)	Germination	C	4.5 ± 0.9 a	8.5 ± 0.5 a	1.2 ± 0.3 a	7.2 ± 3.7 a
		-0.25	5.2 ± 1.3 a	13.5 ± 1.7 a	3 ± 0.4 ab	5.2 ± 1.4 a
		-0.5	10.7 ± 2.3 ab	25.7 ± 0.3 b	6.7 ± 2.9 ab	11.5 ± 4.5 a
		-0.75	18.7 ± 3.7 b	15.5 ± 3.6 a	7.2 ± 1.7 ab	9.7 ± 2.3 a
		-1	n.g.	10.5 ± 2.0 a	10.2 ± 1.5 b	0.7 ± 0.5 a
	Recovery	-0.25	-	4.7 ± 3.7	-	-
		-0.5	2.7 ± 1.7	5 ± 2.3	-	1.0
		-0.75	1.0	7.7 ± 1.3	1.0	5 ± 2.6
		-1	2.25 ± 0.3	5 ± 1.7	2.75 ± 1.1	6 ± 2.1
SE	Germination	C	20.9 ± 1.6 a	48.8 ± 3.9 c	76 ± 6.2 c	67.4 ± 2.9 b
		-0.25	48.2 ± 3.9 a	14.3 ± 3.9 ab	28.1 ± 14.0 ab	81 ± 3.0 b
		-0.5	26.6 ± 7.4 a	8.1 ± 1.5 a	74 ± 2.2 c	23.7 ± 8.2 a
		-0.75	23.2 ± 10.9 a	13.1 ± 1.8 a	57.2 ± 2.9 bc	22.5 ± 3.0 a
		-1	n.g.	28.7 ± 5.1 b	10.2 ± 0.9 a	37.5 ± 23.9 b
	Recovery	-0.25	-	83.3 ± 16.7	-	-
		-0.5	83.3 ± 16.7	77.7 ± 14.7	-	66.7 ± 16.7
		-0.75	90.7 ± 4.9	51.7 ± 6.4	100.0	84.6 ± 8.5
		-1	50.7 ± 9.6	72.9 ± 4.7	53.6 ± 13.4	77.8 ± 7.7
GI	Germination	C	14 ± 0.7 d	8.1 ± 0.6 d	16.4 ± 0.5 d	17.8 ± 1.3 d
		-0.25	8.5 ± 0.2 c	5.3 ± 0.2 c	9.4 ± 0.4 c	10.1 ±0.1 c
		-0.5	5.3 ± 0.2 b	1.8 ± 0.1 b	8.2 ± 0.2 c	6.4 ± 0.6 b
		-0.75	2.2 ± 0.3 a	1 ± 0.2 ab	6.7 ± 0.1 b	0.9 ± 0.2 a
		-1	n.g.	0.3 ± 0.04 a	2 ± 0.1 a	0.1 ± 0.03 a
	Recovery	-0.25	-	0.2 ± 0.1	-	-
		-0.5	1.0 ± 0.03	1.6 ± 0.1	-	1.3 ± 0.2

	-0.75	8.5 ± 1.4	4.1 ± 0.3	1.5 ± 0.5	15 ± 1
	-1	16.0 ± 1.4	4.1 ± 0.4	5.8 ± 0.7	20.5 ± 1.3
